# Oxygen Inequity in the COVID-19 Pandemic and Beyond

**DOI:** 10.9745/GHSP-D-22-00360

**Published:** 2023-02-28

**Authors:** Madeline Ross, Sarah K. Wendel

**Affiliations:** a Department of Emergency Medicine, University of Colorado School of Medicine, Aurora, CO, USA.; b Department of Emergency Medicine, University of Virginia School of Medicine, Charlottesville, VA, USA.

## Abstract

Our review of recent publications on medical oxygen availability during the COVID pandemic underscores the urgent need to prevent unnecessary morbidity and mortality resulting from inequitable access to supplemental medical oxygen therapy.

## Key Messages

- The longstanding inequity in access to supplemental medical oxygen therapy became particularly critical in the face of the COVID-19 pandemic.
- Supplemental medical oxygen is an important indicator of the success of the global pandemic response, given that it is the single most important medication for treating COVID-19.
- Improvements in medical oxygen supply must be prioritized to prevent unnecessary morbidity and mortality.
- Expedited solutions must be balanced with locally led and context-specific sustainability planning that consider durable, meaningful change in oxygen sourcing and supply infrastructures.

## INTRODUCTION

By the end of 2021, the World Health Organization (WHO) had reported more than 6.2 million deaths worldwide due to COVID-19.^1^ This is likely not an accurate representation of the true attributable global death toll, particularly in low- and middle-income countries (LMICs). Even before the onset of COVID-19, lower respiratory tract infections or pneumonia remained the largest cause of death due to communicable diseases worldwide.^2^ Given that a fraction of these deaths was likely preventable with adequate oxygen therapy, there has been a longstanding but urgent need to assess and radically alter the mechanisms for oxygen supply. Thus, the goal of creating equitable, universal access to the “single most important medicine” for treating COVID-19^3^ has been at the core of the current crisis response and will remain crucial to ultimately achieve the Sustainable Development Goals.^4^

The preexisting deficit of medical oxygen in resource-limited settings has been markedly compounded by the COVID-19 pandemic.^5^^,^^6^ Together, oxygen-requiring pathologies like pneumonia, chronic obstructive pulmonary disease, pulmonary hypertension, asthma, and neonatal respiratory syndrome account for 1.75 million deaths annually in sub-Saharan Africa (SSA).^7^ While needs have varied across epidemiological waves, the WHO estimates that approximately 15%–20% of patients with COVID-19 illness require supplemental oxygen therapy.^8^ Furthermore, a 10-country retrospective analysis in Africa showed that among COVID-19 patients in intensive care units, 50% of patients died without supplemental oxygen.^9^ Not accounting for treatment of other oxygen-requiring conditions, the estimated daily need for oxygen in the treatment of COVID-19 cases in LMICs at the end of 2021 remained at more than 1 million oxygen cylinders per day.^10^ Additionally, many LMICs in SSA have also been disproportionately affected by inequity in access to vaccines and basic medical supplies (e.g., face masks). Given that vaccines lower the risk of virus progression and face masks, social distancing, and other measures slow virus spread, these additional inequities likely intensified the mismatch between oxygen capacity and demand in these contexts. Furthermore, a recent United Nations Development Programme analysis estimates that if low-income countries had the same vaccination rates as high-income countries (HICs), their GDP would have increased by US$16.27 billion in 2021, demonstrating the widening gap across development areas.^11^

And so, adequacy of oxygen supply must also be coupled with training regarding appropriate oxygen use, adequate health care staffing, monitoring equipment, personal protective equipment, vaccination, and other essential interventions to meaningfully address the existing inequities across contexts during the COVID-19 pandemic and beyond. Yet, examination of oxygen supply mechanisms and responses remains a critical indicator for overall health system development.

## OXYGEN SUPPLY MECHANISMS

Official standards require medical grade oxygen to be at least 82% oxygen, significantly more concentrated than the atmospheric partial pressure of 21%.^12^ This concentration of medical-grade oxygen is created at many different scales and with many different methods worldwide.

Currently, most HICs maintain ongoing oxygen supplies through the use of massive commercial air separation unit (ASU) plants that cryogenically distill oxygen into its liquid form.^13^ This liquid oxygen (LOX) can then be stored and delivered by tanker to facilities for most oxygen needs. This supply is often augmented by stores of oxygen cylinders at facilities as backup in case of tank failure.^14^^,^^15^ While medical oxygen can be produced in large quantities by this method, the system requires financial and industrial investment up-front, as well as sufficient infrastructure to transport the highly flammable substance, store it in large tanks at health facilities, and ultimately pipe it to end users within the facilities. Demand for medical oxygen increased substantially in HICs during the pandemic. In some regions of Italy, consumption of oxygen increased by 3 times during the pandemic.^13^ However, partnerships between the commercial suppliers and medical authorities in many HICs have been quick to respond to this surge in demand with rapid installation of new pipelines and storage tanks, as well as training of military and private haulers to upscale delivery personnel.^16^

Unsurprisingly, the density of LOX-producing ASU plants in most LMICs is significantly lower. In fact, a 2019 report cited that only 10 countries comprised more than 80% of the total exports of LOX worldwide.^17^ And although there are 50 cryogenic ASUs producing LOX in SSA, most products are utilized in industries such as mining, not health care, and requisite built infrastructure for use in health care facilities remains lacking.^7^ Additionally, transportation resources are limited to get LOX from production sites to health care settings, as this requires large trucks driving flammable material over poorly maintained roads. Some LMICs import medical oxygen; however, high prices have made this option prohibitive.^18^

As such, dependence on this method of oxygen supply is often not feasible, and pressure swing adsorption (PSA) plants are the main alternative plant source. However, the scale of production is smaller: ASUs can produce up to or even more than 5,000 tons per day of oxygen while PSAs and other non-cryogenic air separation methods can typically only produce up to 500 tons per day of oxygen.^19^ Like ASUs, these stand-alone PSA plants create medical-grade oxygen that can be piped directly to patient care areas or compressed and stored in cylinders. Often located on-site at larger health facilities or at strategic locations when commercial, the PSA plants require sizeable up-front financial investment, ongoing maintenance, and a reliable, uninterrupted electrical power source. Traditionally larger and more complex, PSA plants that are condensed, skid-mounted, and easy to install have more recently become available on the market.^13^ However, additional resources are required to keep these plants functional, including workforce capacity and support (including training for both biomedical technicians and health care workers), equipment (including spare parts), and ongoing support with access to experts (e.g., for complex repairs). Given that the WHO has estimated that only 28% of health facilities and 34% of hospitals in SSA have access to reliable electricity, facility-based PSA use may prove challenging in certain contexts.^20^

Currently, much of the oxygen provided at health facilities in LMICs is through the use of cylinders and concentrators due to limitations in availability or prohibitive cost of LOX. Cylinders of oxygen, compressed from either LOX or gas plants, must be transported to end users from central production plants. They are widely regarded as the lowest initial capital cost option and thought to be easier to use than concentrators. Additionally, these can be used during power outages and have minimal maintenance. Though high oxygen flow rates, necessary for the treatment of severe respiratory conditions, can be achieved by the use of cylinders, the supply is exhaustible and requires frequent refills. Furthermore, transportation challenges and restricted patient use (only 1 cylinder per patient, ideally) may outweigh any initial savings.^21^

Currently, much of the oxygen provided at health facilities in LMICs is through the use of cylinders and concentrators due to limitations in LOX availability or its prohibitive costs.

In contrast, concentrators are sources of medical-grade oxygen that can be used directly for end users as long as there is constant electricity and maintenance with which to run them. Concentrators are a mainstay for oxygen provision in many LMICs, as well as for many emergency response efforts, given that they are relatively cheap, easy to transport/maintain, and require low to moderate amounts of training to use.^21^ However, concentrators do have noteworthy problems. First, compared to conventional PSA plants producing flow rates of 8–2,500 liters per minute of oxygen at pressures of 50 psi, concentrators typically deliver flow rates of 3–12 liters per minute of oxygen with pressures of less than 19 psi.^22^ Therefore, the flow rates and delivery pressures of concentrators are often too low for treatment of severe disease, and they are not scalable for the treatment of many patients at once.^13^ A study of 11 health care facilities in rural Kenya showed that facilities had a median of 7 power outages per week with a median duration of 17 minutes, with the longest outage being more than 6 days.^23^ It was also estimated that 32% of patients had oxygen interruption on average of 11 minutes (interquartile range: 9–20), and backup cylinders were only used in 28% of cases.^23^ Beyond electrical interruptions, the efficacy of concentrator use can be limited by improper or unavailable maintenance. A recent publication evaluated the oxygen equipment in southwest Nigeria and found that of 57 concentrators, only 2 were “fit for use,” with most blowing room air and not medical oxygen.^24^

## THE CURRENT STATE OF OXYGEN SUPPLY IN SSA

More than 10 years ago, in the wake of the influenza A (H1N1) pandemic, a survey of 12 countries in SSA using the WHO Tool for Situation Analysis to Assess Emergency and Essential Surgical Care demonstrated that less than 50% of surveyed facilities had uninterrupted access to oxygen and less than 25% had a functioning oxygen concentrator.^21^ These findings heralded the urgent need for investment and attention in the space to prepare for the next influenza-like pandemic.^21^

Although data on country-specific oxygen capacity and therapy delivery remains largely lacking in LMICs, there are a few reports that shed light on the current state. It is essential to note that most of these reports only assessed if there was oxygen available, not the quality of the oxygen supply being utilized. Therefore, it is possible that even these reports on availability overestimate true measures of access. Unfortunately, the evidence suggests that the oxygen gap remains particularly wide for LMICs in SSA.^25^

The evidence suggests that the oxygen gap remains particularly wide for LMICs in SSA.

Service provision assessment responses from the Democratic Republic of the Congo for 2016–2017, Senegal for 2014–2017, Malawi for 2013–2014, and Tanzania for 2014–2015 found that less than half of facilities (secondary-level facilities, health centers, and clinics) had both continuous power and available oxygen.^26^^,^^27^ Researchers also noted that less than 7% of facilities responded to surveys in these countries, raising concerns that oxygen may be even more limited than suggested in their findings.^28^ Similarly, the Ethiopia Federal Ministry of Health reported in 2016 that only 62% of hospitals in the country had a sufficiently equipped/filled oxygen cylinder or functional concentrator available for consumption in the inpatient pediatric department and only 14% had staff properly trained to provide oxygen therapy. Approximately 90% of health facilities assessed had electrical power interruptions of 2 hours or more in the week prior to assessment. Of the hospitals providing oxygen therapy, more than 90% reported use of cylinders and/or concentrators, while only 2% reported use of plant-sourced oxygen.^28^

A more recent 2021 PATH report primarily conducted in Kinshasa, Democratic Republic of the Congo, found that 40% of 93 surveyed facilities—including tertiary-level hospitals, provincial hospitals, general hospitals, and health centers—still did not offer oxygen therapy.^29^ When investigated further, these data also showed that 13 of the 47 facilities with intensive care units did not have oxygen therapy available.^29^ This particular finding underscores that even many facilities caring for critically ill patients lack medical oxygen for treatment.^28^

## THE GLOBAL RESPONSE TO THE OXYGEN CRISIS

In the wake of renewed attention to this longstanding global emergency during the current COVID-19 crisis, many large multinational organizations have integrated oxygen-specific planning into their overall pandemic response strategies. However, the success in implementation and effectiveness of these responses is yet to be fully known.

In 2021, the COVID-19 Oxygen Emergency Taskforce was created as a collaboration between 9 organizations, including the WHO, under the broader umbrella of the Access to COVID-19 Tools Accelerator Therapeutics Pillar. The task force was established with the goal of “measuring acute and longer-term oxygen needs in LMICs; connecting countries to financing partners for their assessed oxygen requirements; and supporting the procurement and supply of oxygen, along with related products and services.”^30^ Additionally, secondary goals were to address “the need for innovative market-shaping interventions” and advocacy efforts regarding oxygen access.^30^ The task force selected 20 countries to target in initial efforts by matching national needs with World Bank and Global Fund financing.^30^ Under this task force, the Clinton Health Access Initiative and Unitaid spearheaded groundbreaking, cooperative memoranda of understanding with 2 major global LOX manufacturers, Air Liquide and Linde, to increase access to medical oxygen in LMICs.^31^ However, this agreement is nonbinding, and the impact will require further investigation.

UNICEF describes its response as 3-pronged: direct donations, oxygen system building, and innovation fast-tracking. Since the pandemic began, UNICEF launched the Oxygen Therapy Project to provide national governments with practical tools for building systems with planning and procurement guidance manuals, as well as technical support for installing equipment and training staff.^12^ For example, in Sierra Leone, UNICEF deployed biomedical engineers to complete a stalled installation of an oxygen plant in a strategic facility and solicited PSA suppliers to install and maintain 3 more plants across the country.^32^ The project is coupled with UNICEF’s preexisting SPRINT (Scaling Pneumonia Response InnovaTions) Project that began in 2018 but has been reemphasized during the pandemic. The SPRINT Project provides country-level triaging tools to assess capacity and bottlenecks in critical interventions for treating respiratory illness. In countries like Ghana and Senegal, where SPRINT had been piloted before 2020, key pieces of the oxygen supply and procurement network had already been addressed, allowing increased ability to withstand the surges in demand.^33^ In addition, UNICEF has organized several workgroups to accelerate the development of technologies for use in low-resource environments, such as handheld pulse oximeters, more durable concentrators, and an all-in-one PSA kit called an “Oxygen Plant in a Box.”^12^

Similarly, PATH had established a robust Oxygen Delivery Toolkit before the onset of the COVID-19 pandemic but has since rapidly expanded its available resources. The toolkit includes overviews of oxygen delivery business models, respiratory care procurement options, a list of sub-Saharan African respiratory care equipment distributors, market reports, training resources, and facility assessment resources, as well as many global and country-specific policies, protocols, and tools that can be readily adapted.^34^

Médecins Sans Frontières (MSF) has approached oxygen procurement in a targeted, context-specific fashion true to their embedded response tactics in over 700 health care facilities, 900 retirement and nursing homes, and 200 sheltering facilities worldwide.^3^ For example, in the Democratic Republic of the Congo, MSF has utilized novel approaches to cylinder-based oxygen delivery at a large COVID-19 treatment facility in Kinshasa by creating a central bank of cylinders that prevents time-intensive bedside tank changes and decreases the incidence of hypoxemia in patients secondary to depleted oxygen supplies going unnoticed.^3^ Additionally, MSF has led efforts to identify multiple suppliers of medical-grade oxygen in the region to ensure continuous sourcing.^3^

Beyond macro-level response strategies, many organizations have given direct donations to augment oxygen capacity immediately. For example, UNICEF announced donations of over 20,000 concentrators, 42,000 accessories (pulse oximeters, flow splitters, etc.), and 1 million consumables (nasal cannulas, tubing, etc.) across 94 countries between January 2020 and May 2021.^12^ The United Kingdom and WHO provided South Sudan with 160 oxygen concentrators, while also pledging to build a new oxygen plant at a referral teaching facility.^35^ Similarly, the U.S. Agency for International Development donated 4 oxygen plants and 28 oxygen concentrators to Ghana.^36^

Many countries have also initiated national programs alongside the acceptance of directed donations from international partners to address the ongoing oxygen shortfall. In Nigeria, for example, the national government pledged US$16.9 million to build oxygen plants at 38 sites. The government is also utilizing the Nigerian Air Force to support oxygen transportation efforts in the interim.^37^

Many countries have initiated national oxygen programs to address the ongoing oxygen shortfall.

Other countries are benefiting from public-private partnerships and localized efforts. Ethiopia’s Amhara Region Oxygen Centre, a joint effort between the Federal Ministry of Health, the Amhara Regional Health Bureau, General Electric, and Assist International, is now providing the region with reliable oxygen from 2 plants commissioned just before the pandemic.^38^ In Kenya, Hewatele, a private company founded in 2014, seeks to lower the cost of oxygen to health care facilities by investing in small oxygen plants in rural areas.^39^ Since the pandemic began, Hewatele has promised a new US$15 million investment to expand its “milkman” model of oxygen delivery and to build a LOX plant.^40^

## CHALLENGES

Despite these efforts, a variety of barriers have hindered rapid upscaling of oxygen delivery in LMICs to date. With such preexisting scarcity of local oxygen production in LMICs in SSA, it is no surprise that cost has dramatically risen during the pandemic. Stein et al. describe the cost of oxygen in SSA as prohibitive, noting that the cost of oxygen typically used by 1 adult patient in 24 hours can reach up to US$100 in Somalia.^27^ MSF has cited similar cost increases across other global response contexts, noting that the private sector controls most oxygen plants in countries where they are working.^3^ This pattern raises concern regarding oversight mechanisms to prevent price-gouging in times of crisis in monopoly markets.

Although there are ongoing decentralization efforts to increase oxygen access at the provincial and district levels in many SSA countries, both the funding mechanisms and actual installation of oxygen plants can be slow. These delays are further compounded in contexts where staff need extensive training to run and maintain plants, as well as to transport the oxygen once produced. Even once new plants are built, the relatively low overall density of plants requires oxygen to be transported longer distances than in most HICs. Unfortunately, basic infrastructure such as road maintenance, inconsistent fuel availability and cost, and high rates of traffic accidents may prevent rapid, safe, and reliable delivery.^41^

While some direct donations of oxygen resources have included disproportionate numbers of ventilators or other invasive devices, which demonstrates a lack of understanding of the realities of many resource-limited environments, most have focused on shipments of oxygen concentrators. However, oxygen concentrators often require uninterrupted electricity to run, which isn’t always accessible, and may be further affected by temperature and humidity. These response efforts are also largely dependent on external donor funding. While capital investments in equipment and installation are critical, not all have included intentional planning for the inevitable workforce and logistical infrastructure needed for ultimate service delivery. As a result, some critics are calling for improved efforts to “align global-development-partner-supported commodity procurement with country realities to enable delivery of the highest value interventions.”^42^

## CONCLUSION

With the acknowledgment that oxygen supply cannot be the singular focus of the response to meaningfully impact person-centered outcomes in this pandemic and the next, it is a valuable indicator for the success of the global response, given the consensus that it is the single most important medication for the treatment of COVID-19.

For too long, and as demonstrated in recent Ebola virus disease outbreaks, valid criticism has centered on the Global North’s focus on humanitarian efforts to prevent the spread of infectious diseases at the expense of therapeutic interventions and strengthening of health care systems. The global community has a collective responsibility to support local and national partners whose oxygen capabilities have been overwhelmed by COVID-19 and other cascading crises in a timely, effective, equitable, collaborative, and responsible manner that strengthens local capacity.

The global community has a collective responsibility to support local and national partners in timely, effective, equitable, collaborative, and responsible manner that strengthens local capacity for those whose oxygen capabilities have been overwhelmed.

As the pandemic lingers and parts of the world begin to enter recovery phases in the disaster cycle, long-term strategic response and recovery must ensure meaningful change in oxygen sourcing and supply infrastructures, such that medical oxygen can continue to be reliably available for all patients requiring it in the current crisis, at baseline for a multitude of other conditions, and in future disasters worldwide.^43^

All nations must prioritize these efforts to increase oxygen availability, but these immediate interventions must be balanced with sustainability planning that is locally led and context- and resource-specific. Equal emphasis on the accompanying training, technology, infrastructural, governance, and financial investments will be needed for full realization of universal accessibility to medical oxygen. And so, while the global response to oxygen provision during the COVID-19 pandemic has thus far proved insufficient, the global call to action spurred by the pandemic must be heeded to avoid repeating the inaction that followed the H1N1 pandemic.

## Notes

**Peer Reviewed**

**First published online**: February 10, 2023.
